# The carbon-concentrating mechanism of the extremophilic red microalga *Cyanidioschyzon merolae*

**DOI:** 10.1007/s11120-023-01000-6

**Published:** 2023-02-13

**Authors:** Anne K. Steensma, Yair Shachar-Hill, Berkley J. Walker

**Affiliations:** 1 Department of Plant Biology, Michigan State University, East Lansing, MI USA; 2 Michigan State University - Department of Energy Plant Research Laboratory, Michigan State University, East Lansing, MI USA

**Keywords:** Carbon-concentrating mechanisms, *Cyanidioschyzon merolae*, Cyanidiales, Photosynthesis, Extremophiles, Gas-exchange

## Abstract

*Cyanidioschyzon* *merolae* is an extremophilic red microalga which grows in low-pH, high-temperature environments. The basis of *C. merolae*’s environmental resilience is not fully characterized, including whether this alga uses a carbon-concentrating mechanism (CCM). To determine if *C. merolae* uses a CCM, we measured CO_2_ uptake parameters using an open-path infra-red gas analyzer and compared them to values expected in the absence of a CCM. These measurements and analysis indicated that *C. merolae* had the gas-exchange characteristics of a CCM-operating organism: low CO_2_ compensation point, high affinity for external CO_2_, and minimized rubisco oxygenation. The biomass δ^13^C of *C. merolae* was also consistent with a CCM. The apparent presence of a CCM in *C. merolae* suggests the use of an unusual mechanism for carbon concentration, as *C. merolae* is thought to lack a pyrenoid and gas-exchange measurements indicated that *C. merolae* primarily takes up inorganic carbon as carbon dioxide, rather than bicarbonate. We use homology to known CCM components to propose a model of a pH-gradient-based CCM, and we discuss how this CCM can be further investigated.

**Supplementary Information:**

The online version contains supplementary material available at 10.1007/s11120-023-01000-6.

## Introduction

Phototrophs have an array of strategies to combat photosynthetic inefficiencies that arise from the competition between carbon dioxide (CO_2_) and molecular oxygen (O_2_) fixation by rubisco (EC 4.1.1.39), the initial enzyme of C_3_ photosynthesis. Some photosynthetic organisms are capable of increasing CO_2_ fixation efficiency by operating carbon-concentrating mechanisms (CCMs), which help inhibit rubisco’s oxygenase activity by increasing the CO_2_ concentration around rubisco. Aquatic organisms are notable for their widespread and varied CCMs, which enable them to be highly photosynthetically productive despite the limited solubility and slow diffusion of CO_2_ in aqueous environments (Griffiths et al. 2017). In particular, aquatic organisms with CCMs predominately operate various biophysical CCMs, which are CCMs that involve movement of dissolved inorganic carbon (DIC) species, rather than involving the fluxes of carbon through organic carbon metabolism which characterize the biochemical CCMs of C_4_ and CAM plants (Barrett et al. 2021). Expanding our knowledge of this CCM diversity is essential to understanding how organisms resist environmental challenges and may reveal new approaches to overcoming biotechnological challenges. For example, the engineering of aquatic CCMs into C_3_ crop plants represents a possible avenue for increasing agricultural productivity (Price et al. 2011; McGrath and Long 2014; Meyer et al. 2016; Rae et al. 2017; Hennacy and Jonikas 2020).

*Cyanidioschyzon merolae* is a photosynthetic aquatic organism which overcomes substantial barriers to inorganic carbon (Ci) acquisition and fixation. The common laboratory strain of this extremophilic red microalga, *C. merolae* 10D, was first isolated from the Italian volcanic caldera Campi Flegrei and is optimally cultured at pH 1.5–2.5 and temperature 42–45 °C (De Luca et al. 1978; Albertano et al. 2000; Miyagishima and Wei 2017). Ci concentrations in *C. merolae*’s natural environment likely span a significant range, as Campi Flegrei’s carbon output varies over time, and the waters of this volcanic system vary considerably in Ci content (Venturi et al. 2017; Chiodini et al. 2021). In the laboratory, ambient concentrations of CO_2_ are sufficient for cultivation of *C. merolae*. Supplying 5% CO_2_ in combination with increased light intensity accelerates *C. merolae*’s growth, but under constant light this alga maintains similar growth rates when transitioned between 0.04% and 5% CO_2_ (Minoda et al. 2004; Rademacher et al. 2016; Miyagishima and Wei 2017). High-temperature and low-pH conditions limit CO_2_ solubility and restrict the accumulation of dissolved Ci species other than CO_2,_ which readily outgasses from aqueous environments (Oesterhelt et al. 2007). The resulting Ci scarcity likely presents an obstacle to growth of *C. merolae*, as this alga appears to depend on phototrophy. The only known method for heterotrophic culture of wild-type *C. merolae* requires supplying cells with glycerol concentrations far higher than are typical in natural or laboratory conditions, and the resulting heterotrophic growth is slow and ceases after 6–7 cell divisions (Moriyama et al. 2015, 2017, 2018; Liu et al. 2020). Accordingly, genomic data suggests that *C. merolae* may have a relatively limited capacity for organic carbon uptake: *C. merolae*’s genome has substantially fewer putative carbohydrate transporters and glycerol permeases than the genome of a closely related facultative heterotroph (Weber et al. 2004; Barbier et al. 2005; Fujiwara et al. 2019). How *C. merolae*’s photosynthesis is adapted to its extreme environment, including whether the organism has a CCM, is not well-understood. Most biophysical CCMs involve uptake of the Ci species bicarbonate, which is more easily concentrated within membranes than CO_2_ (Mangan et al. 2016). However, bicarbonate is minimally available at low pH, presenting further challenges to Ci acquisition in *C. merolae*.

In addition to being an interesting system for the study of photosynthesis under extreme conditions, *C. merolae* is experimentally tractable due to its simple cellular and metabolic structures, its highly reduced and completely sequenced genome, and its amenability to molecular techniques (Matsuzaki et al. 2004; Kuroiwa et al. 2017). Further studies of *C. merolae* will expand our knowledge of the phylogenetically remote algae order Cyanidiales, which includes the only eukaryotes and phototrophs known to tolerate the conditions of acidic sulfur-rich geothermal springs (Miyagishima et al. 2017; Stadnichuk and Tropin 2022). This unique environmental resilience suggests that Cyanidiales hold promise for production of biofuels and other high-value algal products. For example, Cyanidialean algae like *C. merolae* and *Galdieria* species are of biotechnological interest in part because they can grow in wastewater and in environments which inhibit the growth of the culture-contaminating organisms that commonly plague aquaculture (Varshney et al. 2015; Sato et al. 2017; Lang et al. 2020; di Cicco et al. 2021). Understanding mechanisms by which Cyanidiales increase carbon capture efficiency may thus have biotechnological value.

CCMs by definition substantially accumulate intracellular inorganic carbon, as is detectable in *C. merolae* (Zenvirth et al. 1985)*.* However, the existence of the CCM in *C. merolae* is uncertain due to the necessity of a functional photorespiratory pathway in this alga and to variation in this alga’s CO_2_ compensation point with O_2_ concentration (Rademacher et al. 2016; Parys et al. 2021). Here we show that *C. merolae* exhibited CO_2_ uptake characteristics that are unlikely to be explained by rubisco’s kinetic properties alone. In particular, we show that *C. merolae* exhibited the gas-exchange features which are outcomes of all CCMs: low CO_2_ compensation point, high affinity for external CO_2_, and minimized rubisco oxygenation. The carbon isotope composition of *C. merolae*’s biomass was also consistent with a CCM. Additionally, our gas-exchange measurements indicated that *C. merolae* primarily takes up Ci as CO_2_, rather than bicarbonate. We use homology to known CCM components to propose a model of a unique pH-gradient-based CCM, and we discuss how this model may be investigated in *C. merolae*.

## Results

### *Cyanidioschyzon merolae* ’s CO2 compensation point, affinity for CO2, and oxygen response are not fully explained by rubisco kinetics of thermophilic red algae

To determine whether *C. merolae* shows gas-exchange features consistent with the operation of a CCM, we measured the cellular CO_2_ compensation point (${\Gamma }_{{CO}_{2}}$) and affinity for CO_2_ (${K}_{{m(CO}_{2})}$) under varying temperature and oxygen conditions. CCMs can boost photosynthetic efficiency beyond what is explainable by an organism’s rubisco kinetics (Raven and Beardall 2003; Giordano et al. 2005). Therefore, we built a quantitative framework for interpreting our gas exchange data by calculating the ${\Gamma }_{{CO}_{2}}$ and ${K}_{{m(CO}_{2})}$ permitted by combinations of the rubisco kinetic parameters of thermophilic red algae, assuming the absence of a CCM or of ribulose 1,5-bisphosphate (RuBP) limitation. Details on these calculations are provided in the Methods section, and Table 1 lists the inputs used for calculations (including values from Fig. S1), parameter definitions, and input values.

**Table 1:** Parameters used in model calculations of gas-exchange parameters from rubisco kinetics

Parameter	Definition	Value(s)	Source(s)
*S*_*c/o*_	The CO_2_/O_2_ specificity of rubisco (ratio of the carboxylase to oxygenase rate when CO_2_ and O_2_ are present at equal concentrations), here presented in molar/molar (liquid-phase) form	224.6 (*Cyanidium caldarium)*	Uemura et al. (1997); Whitney et al. (2001)
		238.1 (*Galdieria partita)*	
		166 (*Galdieria sulphuraria)*	
*K*_*c*_	The Michaelis–Menten constant of rubisco for CO_2_	6.7 µM (*Cyanidium caldarium)*	Uemura et al. (1997); Whitney et al. (2001)
		6.6 µM (*Galdieria partita)*	
		3.3 µM (*Galdieria sulphuraria*)	
*K*_*o*_	The Michaelis–Menten constant of rubisco for O_2_	374 µM (*Galdieria sulphuraria*)	Whitney et al. (2001)
*R*_*L*_* / V*_*cmax*_	The ratio of CO_2_ loss in the light, *R*_*L,*_ to rubisco’s maximal rate of carboxylation, *V*_*cmax*_	0.13 (40 °C)	This study; ratio of cellular* R*_*L*_ to cellular maximal assimilation rate, *A*_*max*_, as determined from light response curve (Fig. S1)
		0.07 (30 °C)	
*O*	Chloroplastic O_2_ concentration	20 µM (2% O_2_ atmosphere, 40 °C)	This study; Henry’s Law estimates
		209 µM (21% O_2_ atmosphere, 40 °C)	
		398 µM (40% O_2_ atmosphere, 40 °C)	
		251 µM (21% O_2_ atmosphere, 30 °C)	
*H*_*298.15*_	Standard-temperature Henry’s law constant	0.035 mol/(kg*bar) (CO_2_)	NIST
		0.0012 mol/(kg*bar) (O_2_)	
$\frac{-{\Delta }_{sol}H}{R}$	Temperature dependence constant used to adjust standard-temperature Henry’s law constants	2400 K (CO_2_)	NIST
		1700 K (O_2_)	
*Q*_*10*_	Temperature coefficient used to calculate how a parameter’s value would respond to 10 °C temperature shifts	0.60 (*Q*_*10*_ (25 °C) for *S*_*c/o*_ values in the liquid phase)	von Caemmerer (2000); Galmés et al. (2016)
		0.62 (*Q*_*10*_ (35 °C) for *S*_*c/o*_ values in the liquid phase)	
		2.24 (*Q*_*10*_ (25 °C) for *K*_*c*_ values in µbar)	
		1.63 (*Q*_*10*_ (25 °C) for *K*_*o*_ values in mbar)	

**Fig. 1:**
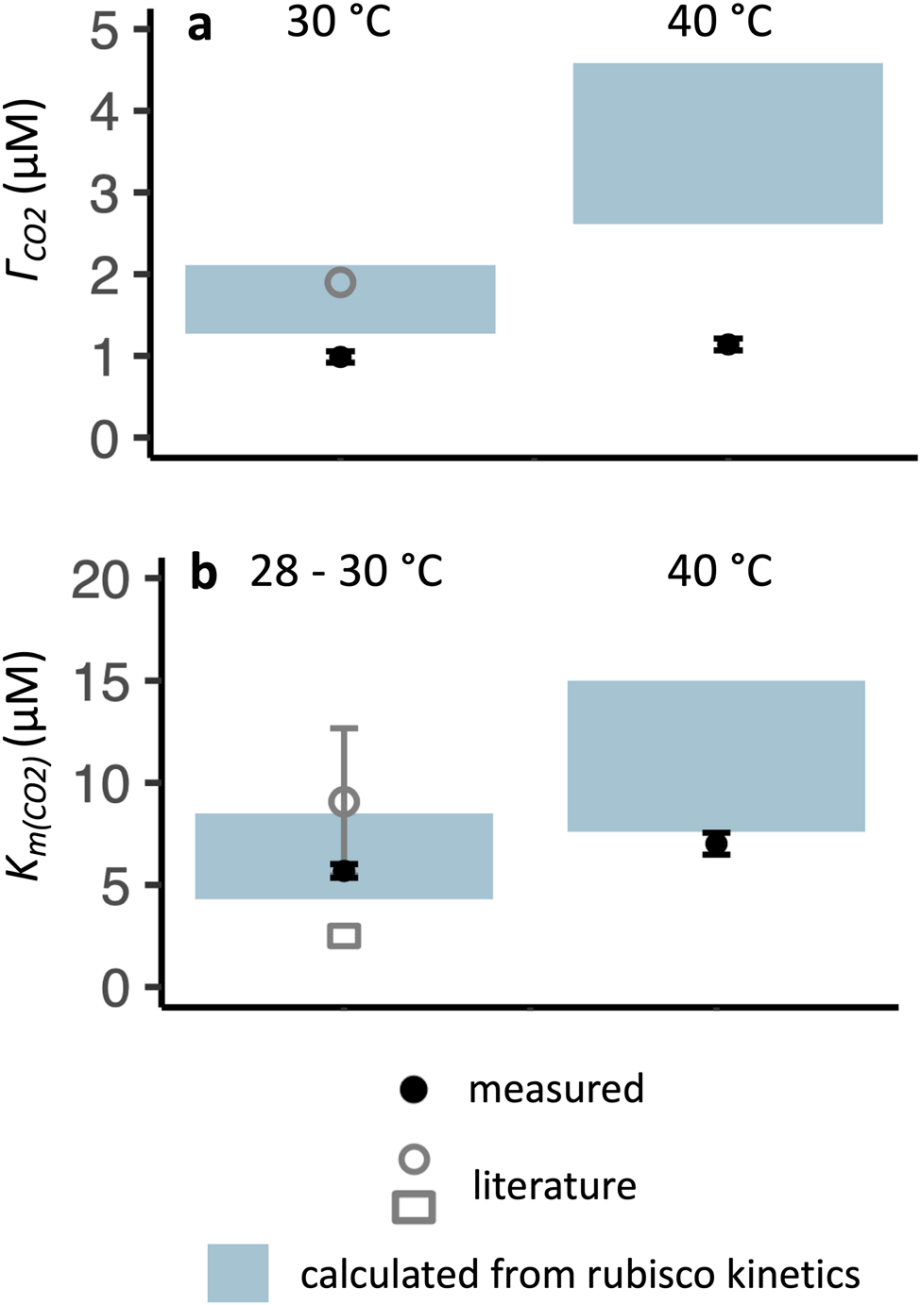
Comparison of gas exchange parameters calculated from rubisco kinetics of thermophilic red algae (shaded areas) to measured gas exchange parameters (black solid points, means of *n* = 3 replicates, with error bars indicating ± 2 SEs), and to literature values (gray open shapes, presented as ranges or as mean ± 2 SEs when available). The parameters examined are **a** ${\Gamma }_{{CO}_{2}}$ and **b** ${K}_{{m(CO}_{2})}$. In **b**, the measured and literature values represent the cellular ${K}_{{m(CO}_{2})}$, while the calculated values represent the rubisco *K*_*c*_. The literature data sources are Zenvirth et al. (1985); Rademacher et al. (2016); Parys et al. (2021), which report oxygen evolution data across external pH 1.5–7.5, oxygen evolution data from cells grown under 5% CO_2_ and exposed to ambient CO_2_ concentrations for 24 h, and a compensation point measurement at 21% O_2_, respectively

Measured ${\Gamma }_{{CO}_{2}}$ values were lower than the calculated ranges, meaning that net carbon assimilation was able to occur at lower CO_2_ concentrations than would be expected in the absence of a CCM. Additionally, rubisco CO_2_ affinity (*K*_*c*_) values (once adjusted for temperature) could be compared to a measured cellular ${K}_{{m(CO}_{2})}$, since *C. merolae*’s carbon assimilation showed a Michaelis–Menten-like response to CO_2_ availability (Fig. S2). Measured cellular ${K}_{{m(CO}_{2})}$ values overlapped only with the temperature-corrected *K*_*c*_ values from *Galdieria sulphuraria* rubisco, which are the lower bounds of the calculated rubisco ${K}_{{m(CO}_{2})}$ ranges (Fig. 1). Literature values of ${K}_{{m(CO}_{2})}$ and ${\Gamma }_{{CO}_{2}}$ had varying degrees of overlap with our calculated values (Fig. 1).

CCMs minimize rubisco oxygenation and thus reduce the response of characteristics of CO_2_ assimilation to oxygen. For example, in the extreme case of an organism with a CCM that completely inhibits rubisco’s oxygenase activity, there would be no oxygen sensitivity of CO_2_ assimilation parameters affected by rubisco oxygenation (such as ${\Gamma }_{{CO}_{2}}$). In the case of an organism with an imperfect but functional CCM, the oxygen response of ${\Gamma }_{{CO}_{2}}$ would be reduced compared to the oxygen response calculated from rubisco kinetics, and this is what appears to have occurred in *C. merolae*. Across all tested oxygen concentrations (2%, 21%, 40%), ${\Gamma }_{{CO}_{2}}$ had a lower value than that calculated from rubisco kinetics, and additionally ${\Gamma }_{{CO}_{2}}$ had a shallower oxygen-response slope than calculated from the rubisco kinetics. The measured slope was 5425 ± 1396 pM CO_2_/μM O_2_ (mean ± 2 SEs, *n* = 6) while the slopes calculated from rubisco kinetics were 7046–11,153 pM CO_2_/μM O_2_.

**Fig. 2:**
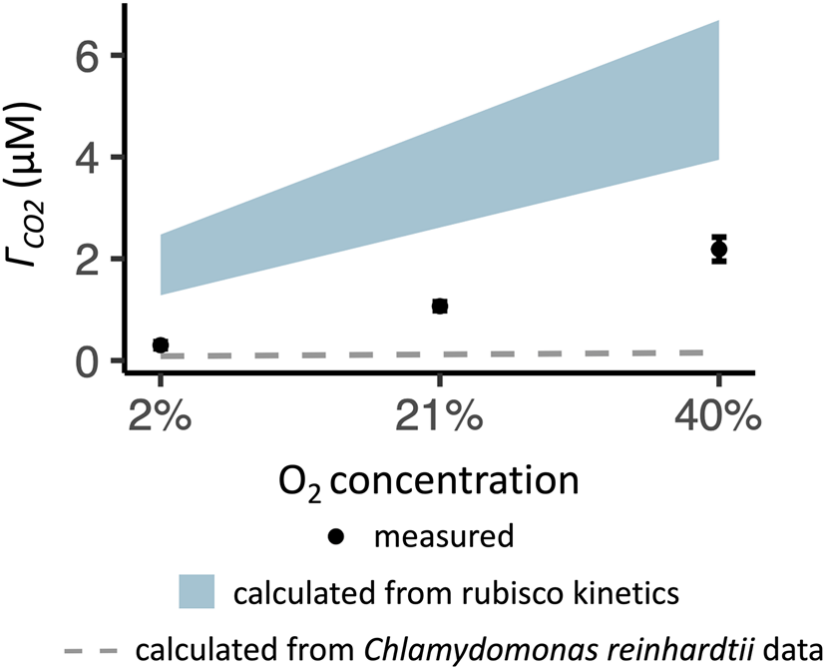
Oxygen response of ${\Gamma }_{{CO}_{2}}$ in *C. merolae.* The measured ${\Gamma }_{{CO}_{2}}$ (points mean ± 2 SEs, 6 total samples in a gas-exchange system supplied with 21% O_2_ and 2% or 40% O_2_) is compared to compensation points calculated from the rubisco kinetics of thermophilic red algae (shaded areas) or from published data on oxygen and temperature response of *C. reinhardtii *(dashed lines). *C. reinhardtii* data is from Coleman and Colman (1980)

While these comparisons between measured and calculated ${\Gamma }_{{CO}_{2}}$, ${K}_{{m(CO}_{2})}$, and oxygen response of ${\Gamma }_{{CO}_{2}}$ were consistent with the presence of a CCM, the ${\Gamma }_{{CO}_{2}}$ values and oxygen-response slope of ${\Gamma }_{{CO}_{2}}$ were larger than those estimated for *C. reinhardtii* under comparable conditions (Fig. 2). The oxygen-response slope of ${\Gamma }_{{CO}_{2}}$ calculated *C. reinhardtii* data was 2 pM CO_2_/μM O_2._

### The stable carbon isotope signature of *C. merolae*’s biomass is isotopically heavier than that of some photosynthetic organisms lacking CCMs

To further investigate whether *C. merolae* shows signatures of a CCM, we determined its biomass δ^13^C. *C. merolae*’s biomass δ^13^C is heavier than is typical for multiple classes of organisms without CCMs (many C_3_ plants, as well as macroalgae and seagrasses that likely do not uptake bicarbonate) (Table 2). However, it is isotopically lighter than literature values for organisms with CCMs (C_4_ plants, CCM-containing microalgae, and macroalgae and seagrasses that likely uptake bicarbonate) (Table 2).

**Table 2:** Biomass δ^13^C in *C. merolae* (mean ± 2 SEs, *n* = 4) and benchmark δ^13^C values

Sample type	^δ13^C	Source
Dssolved bicarbonate	~ 0‰	Raven et al. (2002)
Ambient air in laboratory building, evening	− 9.56‰	This study
Marine macroalgae and seagrasses which are likely to uptake bicarbonate	> − 10‰	Raven et al. (2002)
C_4_ plants	− 12 to − 16‰ (usually approximately -14‰)	O’Leary (1988)
Various CCM-containing microalgae	− 15 to − 21‰ (*C. reinhardtii*: − 18 or − 19‰)	Wu et al. (2012); Goudet et al. (2020)
*C. merolae*	− 23.03 ± 0.16‰	This study
C_3_ plants	− 20 to − 37‰ (estimated global average: − 28.5‰)	Kohn (2010)
Marine macroalgae and seagrasses which are unlikely to uptake bicarbonate	< − 30‰	Raven et al. (2002)

### *Cyanidioschyzon merolae* predominantly takes up CO_2_, rather than bicarbonate

**Fig. 3:**
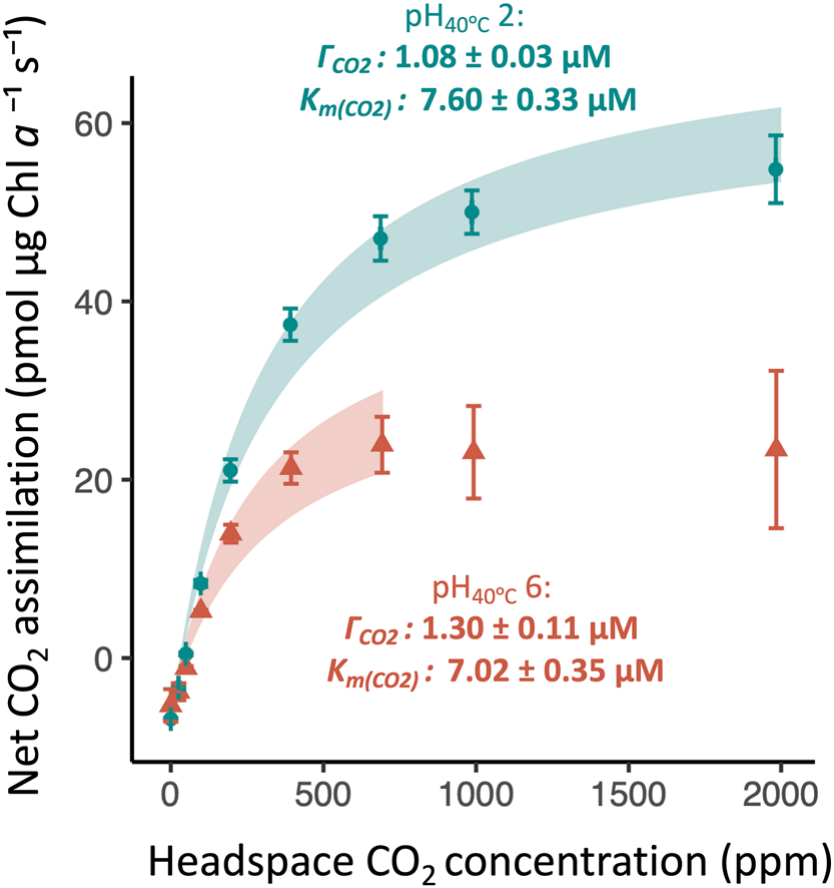
CO_2_ response of cells grown at pH 2.7 and assayed at pH 2 (blue fit and points) or 6 (red fit and triangles) (points and error bars represent mean ± 2 SEs, *n* = 3). A two-parameter Michaelis–Menten curve was fit to all points (pH 2) or to points < 700 ppm (pH 6), and the shaded areas indicate the range of values produced by combining the upper and lower bounds of the calculated Michaelis–Menten parameters (bounds of a 95% confidence interval)

**Fig. 4:**
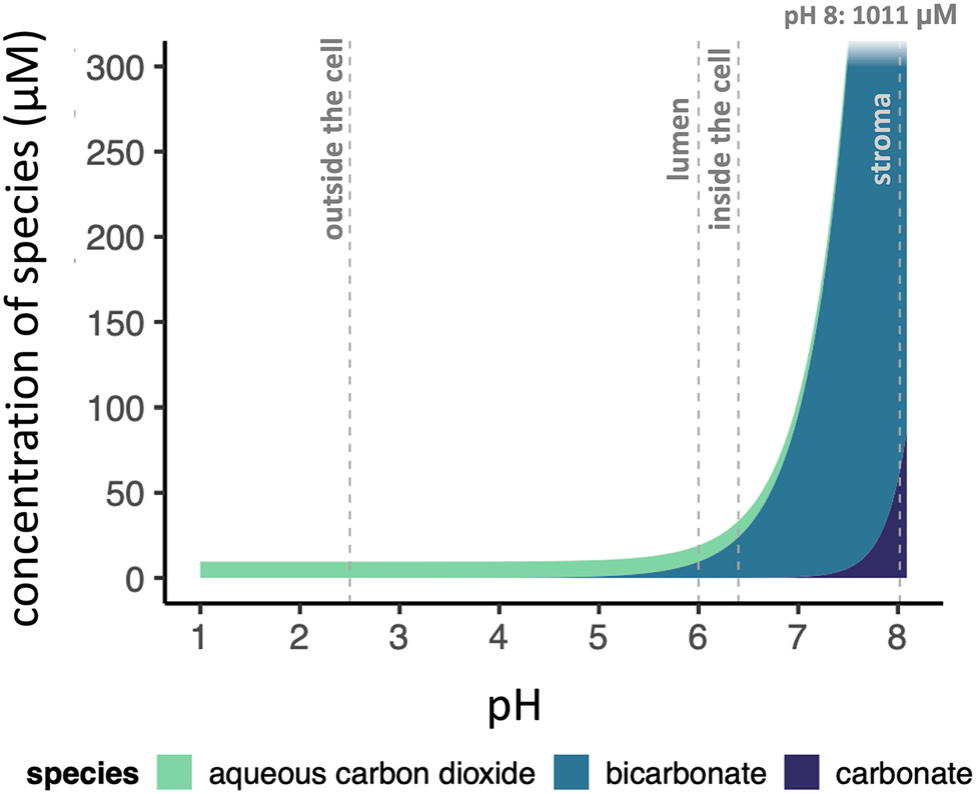
Calculated Ci concentrations in pure water (conditions: 40 °C, 1013.25 hPa, water surface at 400 ppm CO_2_, aqueous CO_2_ concentrations constant across pH). pH benchmarks of interest are indicated, including potential pH conditions outside the cell (the optimal growth pH of *C. merolae*), in the thylakoid lumen (the approximate thylakoid lumen pH of unstressed plants in the light), inside the cell (the average intracellular pH of *C. merolae*), and in the stroma (the stromal pH of spinach chloroplasts in the light). This image is not intended to precisely represent Ci concentrations inside the cell (which could be affected by a number of factors, including various disequilibriums and the presence of other solutes). The y-axis is truncated at 300 µM to provide better visibility of trends at acidic pH. Sources for pH benchmarks: Werdan et al. (1975); Zenvirth et al. (1985); Kramer et al. (1999); Miyagishima and Wei (2017)

To reveal which Ci species is taken up by *C. merolae*, we conducted gas-exchange measurements in cells resuspended at pH 2 or pH 6 (Fig. 3), since pH affects Ci speciation. Bicarbonate is minimally available at pH 2 but comprises approximately half of the Ci pool at pH 6, while aqueous CO_2_ is expected to be similarly available between the two pH conditions. Therefore, if *C. merolae* could take up bicarbonate in addition to CO_2_, its accessible Ci pool would be about twice as large at pH 6 than for pH 2 under the same headspace CO_2_ concentration (Fig. 4). Although there was a decrease with increased pH in ${K}_{{m(CO}_{2})}$ and, perhaps relatedly, a decrease in *A*_*max*_, the decrease in ${K}_{{m(CO}_{2})}$ was not of the magnitude that would be expected if *C. merolae* takes up bicarbonate. Furthermore, there was no decrease in ${\Gamma }_{{CO}_{2}}$ with increased pH (*α* = 0.05; *p* > 0.99 for ${\Gamma }_{{CO}_{2}}$ and ${K}_{{m(CO}_{2})}$ from unpaired *t*-tests with the alternative hypotheses being that the pH 6 parameter means are half of the pH 2 parameter means; *p* = 0.04 for ${K}_{{m(CO}_{2})}$, 0.97 for ${\Gamma }_{{CO}_{2}}$, and 0.0003 for *A*_*max*_ from unpaired *t*-tests with the alternative hypothesis being that the pH 2 parameter means are greater than pH 6 parameter mean). Overall, these gas-exchange results (Fig. 3) indicated that *C. merolae* primarily takes up Ci as CO_2_, even when bicarbonate is available.

### *Cyanidioschyzonmerolae* has homologs to known CCM components

To generate ideas about how the CCM might operate in *C. merolae*, we identified a number of putative homologs to CCM components in *C. merolae*’s genome (Table S1). Notably, we identified two candidates for carbonic anhydrases potentially involved in the CCM (CMI270C, CMT416C) and six candidates for bicarbonate transporters potentially involved in the CCM (CMO283, CMK129C, CMN251C, CMI052C, CMR009C, CMN088C) (Table 3). Some of these candidates have a transcriptional response to CO_2_ (Rademacher et al. 2017) and/or are reciprocal best hits with their query CCM gene, which is consistent with their having a role in the CCM. CCM components with no apparent homologs in *C. merolae* appear to include components that are inessential to or indirectly involved in the algal CCM (e.g., some pyrenoid components, transcriptional regulators, proteins involved in energization of Ci uptake, and additional carbonic anhydrases and Ci transporters) (Table S1).

**Table 3:** Select *C. merolae* loci identified as homologs to CCM-involved genes in other organisms

Gene in *C. merolae* and its annotation	Homologous CCM-involved gene	Percent identity / E score	Predicted localization in *C. merolae*	Transcript abundance response to shift from high to ambient CO_2_ (Rademacher et al. 2017)	Reciprocal best hit
Carbonic anhydrases					
CMI270C, “similar to carbonic anhydrase precursor”	*ecaA*, an α-class carbonic anhydrase of *Synechococcus elongatus* strain PCC 7942	46% / 3e-8	Other, other	0.5-fold at 24 h	Yes
	*CAH1, CAH2,* and *CAH3*, α-type periplasmic or lumenal carbonic anhydrases of *C. reinhardtii*	44 – 46% / 5e-5 – 6e-6			Yes (*CAH3*)
CMT416C, “similar to carbonic anhydrase precursor”	same as above	45% / 2e-7	Thylakoid lumen, chloroplast	65-fold at 3 h, 32-fold at 24 h	Yes
		33–39% / 4e-5–8e-4			Yes (*CAH3*)
Bicarbonate transporters					
CMO283, “unknown ABC anion transporter”	*cmpB,* a gene in the operon encoding the ATP-dependent bicarbonate transporter BCT1 of *Synechocystis PCC6803*	24% / 8e-5	Thylakoid lumen, other	Not significant	Yes
CMK129C, “nitrate ABC transporter ATP-binding protein”	*cmpC* and *cmpD*, genes in the operon encoding the ATP-dependent bicarbonate transporter BCT1 of *Synechocystis* PCC6803	41% / 6e-39 and 3e-43	Thylakoid lumen, chloroplast	Not significant	No
CMN251C, “ATP-binding cassette, sub-family C (MRP), member 1”	*HLA3,* an ATP-binding cassette transporting Ci at the plasma membrane of *C. reinhardtii*	40% / 1e-112	Other, chloroplast	1.9-fold at 3 h	No
CMI052C, “probable sulfate permease”	*bicA,* a sodium-dependent sulfate-permease-type bicarbonate transporter of *Synechocystis* PCC6803	21% / 1e-28	Thylakoid lumen, chloroplast	2.2-fold at 24 h	No
CMR009C, “probable anion transporter”	*PtSLC4-2* and four other *PtSLC4* genes, which are putative sodium-dependent transporters of extracellular bicarbonate of *Phaeodactylum tricornutum*	26 to 48% / 8e-52 to 1e-132	Other	1.6-fold at 24 h	Yes (*PtSLC4-6*)
CMN088C, “hypothetical protein, conserved”	*BST1,* a candidate for a thylakoid bicarbonate transporter of *C. reinhardtii*	26% / 8e-3	Other, secretory pathway	Not significant	No

Localization predictions (TargetP, PredAlgo) of “other” may include targeting to the cytosol, while predictions of “secretory pathway” may include targeting to the endoplasmic reticulum or cytoplasmic membrane (Mori et al. 2016). Otherwise, these tools do not indicate whether proteins may be transported into a membrane. Transcript abundance analysis is sourced from Rademacher et al. (2017), and “significant” transcript responses are those with Bonferroni-corrected *q*-value < 0.01 for comparisons between the high-CO_2_ timepoint and an ambient-CO_2_ timepoint (3 or 24 h after shift to ambient CO_2_). All *C. merolae* genes are top hits of their respective queries, except for CMT416C, which was the second hit of its listed queries. For full homology results and sources for CCM-involved genes, see Table S1

## Discussion

### Gas-exchange and carbon isotope evidence for a CCM in *C. merolae*

*C. merolae*’s ${\Gamma }_{{CO}_{2}}$ and ${K}_{{m(CO}_{2})}$ are consistent with a functioning CCM. *C. merolae* maintained a lower ${\Gamma }_{{CO}_{2}}$ (Fig. 1) and shallower oxygen response of ${\Gamma }_{{CO}_{2}}$ (Fig. 2) than would be expected without a CCM, as evidenced by comparisons to ${\Gamma }_{{CO}_{2}}$ values calculated using the rubisco kinetics from other thermophilic red algae. Furthermore, *C. merolae*’s cellular ${K}_{{m(CO}_{2})}$ is low enough that it overlaps only with the temperature-corrected rubisco *K*_*c*_ measured in *G. sulphuraria* (Fig. 1), which is the lowest-known *K*_*c*_ (Flamholz et al. 2019). A $K_{{m(CO_{2} )}}$ lower than *K*_*c*_ most likely arises from a CCM (Raven et al. 2011).

The biomass δ^13^C of *C. merolae* is also consistent with a CCM, though numerous environmental and physiological factors impact carbon isotope fractionation in biological material (Sharkey and Berry 1985; O’Leary 1988; Raven and Beardall 2003; Hurley et al. 2021). For example, the extracellular δ^13 ^Ci signature varies according to growth environment, and the amount of ^13^C available to *C. merolae* cells would be limited by the low-bicarbonate aqueous growth conditions of this alga*.* Dissolved bicarbonate has an isotopically heavier δ^13^C than CO_2_ (Table 2), and the slower diffusion of isotopically heavier dissolved Ci species (Raven et al. 2002) may be less significant for bicarbonate than for CO_2_, which has a lower molecular weight. Additionally, stable carbon isotopes show an increased solubility of isotopically lighter molecules compared to isotopically heavier molecules, and this effect reduces only very slightly with temperature (Vogel et al. 1970). Despite these constraints on ^13^C availability in *C. merolae*’s growth environment, *C. merolae* had a heavier δ^13^C signature than many photosynthetic organisms which lack a CCM (Table 2). This suggests that *C. merolae*’s biomass δ^13^C is compatible with a stromal environment that limits rubisco’s discrimination against ^13^CO_2_, i.e., the environment produced by a CCM. Interestingly, *C. merolae* had a lighter δ^13^C signature than *C. reinhardtii*, which could result from a more efficient CCM in *C. reinhardtii* (Table 2). *C. merolae*’s CCM form is not sufficiently resolved to determine its CCM efficiency via Ci accumulation ratios; for example, to resolve *C. merolae*’s Ci accumulation ratios with ^14^Ci centrifugation-filtration, it will be necessary to improve understanding of pH and Ci compartmentalization in this alga (Zenvirth et al. 1985). However, the proposition of a CCM in a possibly pyrenoidless alga (Broadwater and Scott 1994; Badger et al. 1998; Albertano et al. 2000; Misumi et al. 2005) which relies on CO_2_ uptake (Fig. 3) is consistent with relatively low Ci accumulation. *C. reinhardtii* cells accumulate Ci up to 40-fold and achieve internal Ci concentrations 5-to-tenfold higher than mutants and morphologically similar species without pyrenoids (Badger et al. 1980; Morita et al. 1998; Meyer et al. 2017). CCMs relying on CO_2_ uptake alone may accomplish only 10-to-15 fold Ci accumulation (Gross 2000). The modest efficiency of *C. merolae*’s CCM is also suggested by the higher ${\Gamma }_{{CO}_{2}}$ and stronger oxygen response of ${\Gamma }_{{CO}_{2}}$ in *C. merolae* as compared to the response of *C. reinhardtii* estimated under comparable conditions (Fig. 2). It will be interesting to further investigate the factors that shaped the evolution of this modest CCM.

*C. merolae*’s rubisco kinetics, though unknown, are unlikely to explain our gas-exchange observations. *C. merolae*’s gas-exchange physiology was not fully explained by the rubisco kinetics of other thermophilic red algae (Figs. 1, 2), which include the highest known rubisco *S*_*c/o*_ and lowest known *K*_*c*_*.* Rubisco kinetics are highly constrained (Flamholz et al. 2019), so it seems improbable that *C. merolae*’s rubisco specificity and affinity would be so high as to explain this alga’s ${\Gamma }_{{CO}_{2}}$ physiology. When we added to our model a temperature-adjusted *S*_*c/o*_ of double the highest temperature-adjusted value and a *K*_*c*_ of half the lowest temperature-adjusted value, we calculated a ${\Gamma }_{{CO}_{2}}$ of 1.3, still slightly higher than *C. merolae*’s ${\Gamma }_{{CO}_{2}}$ of 1.1 μM at low pH, 40 °C, and 21% O_2_ (Fig. 1, Fig. 3). Furthermore, the existence of the CCM in *C. merolae* is supported by direct evidence of intracellular Ci concentration in *C. merolae* (Zenvirth et al. 1985).

Characterization of *C. merolae*’s rubisco will further reveal the drivers of carbon capture efficiency in this alga. *C. merolae*’s rubisco belongs to the subform ID lineage, which is currently associated with high specificity and affinity for CO_2_, especially in organisms without pyrenoids. However, the subform ID lineage has not been extensively characterized (Badger et al. 1998; Giordano et al. 2005; Loganathan and Tsai 2016; Iñiguez et al. 2020), and CCMs may have various evolutionary relationships with rubisco kinetics. For example, CCMs may relax pressure for an organism to improve rubisco’s CO_2_ affinity and specificity (Young et al. 2012; Iñiguez et al. 2020); if such a relaxation occurred in *C. merolae*, it would be all the more indicative of a CCM that *C. merolae*’s cellular ${K}_{{m(CO}_{2})}$ is lower than all known temperature-adjusted rubisco *K*_*c*_ values save one (Fig. 1b).

### A potential pH-gradient-based CCM in *C. merolae* invites further characterization

#### Overview of potential CCM structure in *C. merolae*

Although it appears uncommon for microalgae to lack a CCM (Raven et al. 2011), and knowledge of what is typical for the CCM is still expanding, the apparent presence of a CCM in *C. merolae* is notable because it suggests the presence of a unique mechanism for carbon concentration. *C. merolae* maintained an apparent CCM in a low-bicarbonate external environment, which suggests the presence of a pH-gradient-based concentration of carbon relative to acidic surroundings. Dissolved CO_2_, rather than bicarbonate, appeared to be the primary Ci species taken up by cells (Fig. 3). *C. merolae*’s reliance on CO_2_ is also indicated by the variance of ${K}_{m(Ci)}$ of oxygen evolution with pH, and by ^14^Ci pulse-chase experiments (Zenvirth et al. 1985). *C. merolae*’s reduced *A*_*max*_ at pH 6 or 5.5 compared to pH 2 or 1.5 (Fig. 3, Zenvirth et al. 1985) indicates that pH stress may impair function of acidophiles in near-neutral conditions. However, it seems reasonable that cells adapted and acclimated to low pH would not maintain bicarbonate uptake machinery, as taking up bicarbonate against a large bicarbonate gradient may be prohibitively energetically expensive. Such a bicarbonate gradient (Fig. 4) may also support the CCM. pH-gradient-based CCMs have attracted interest as a possible CCM of acidophilic organisms (Gehl and Colman 1985; Weber et al. 2007; Rademacher et al. 2016). In this type of CCM, the maintenance of near-equilibrium Ci concentrations in a near-neutral cytosol would concentrate carbon relative to acidic surroundings, even if Ci enters the cell only by CO_2_ diffusion. Bicarbonate accumulated in this cytosolic bicarbonate trap could then be transported into the chloroplast. The basic stroma could function as a second bicarbonate trap, as is proposed for *C. reinhardtii* (Fei et al. 2022).

#### *Cyanidioschyzon merolae*’s CCM may depend on active transport

**Fig. 5:**
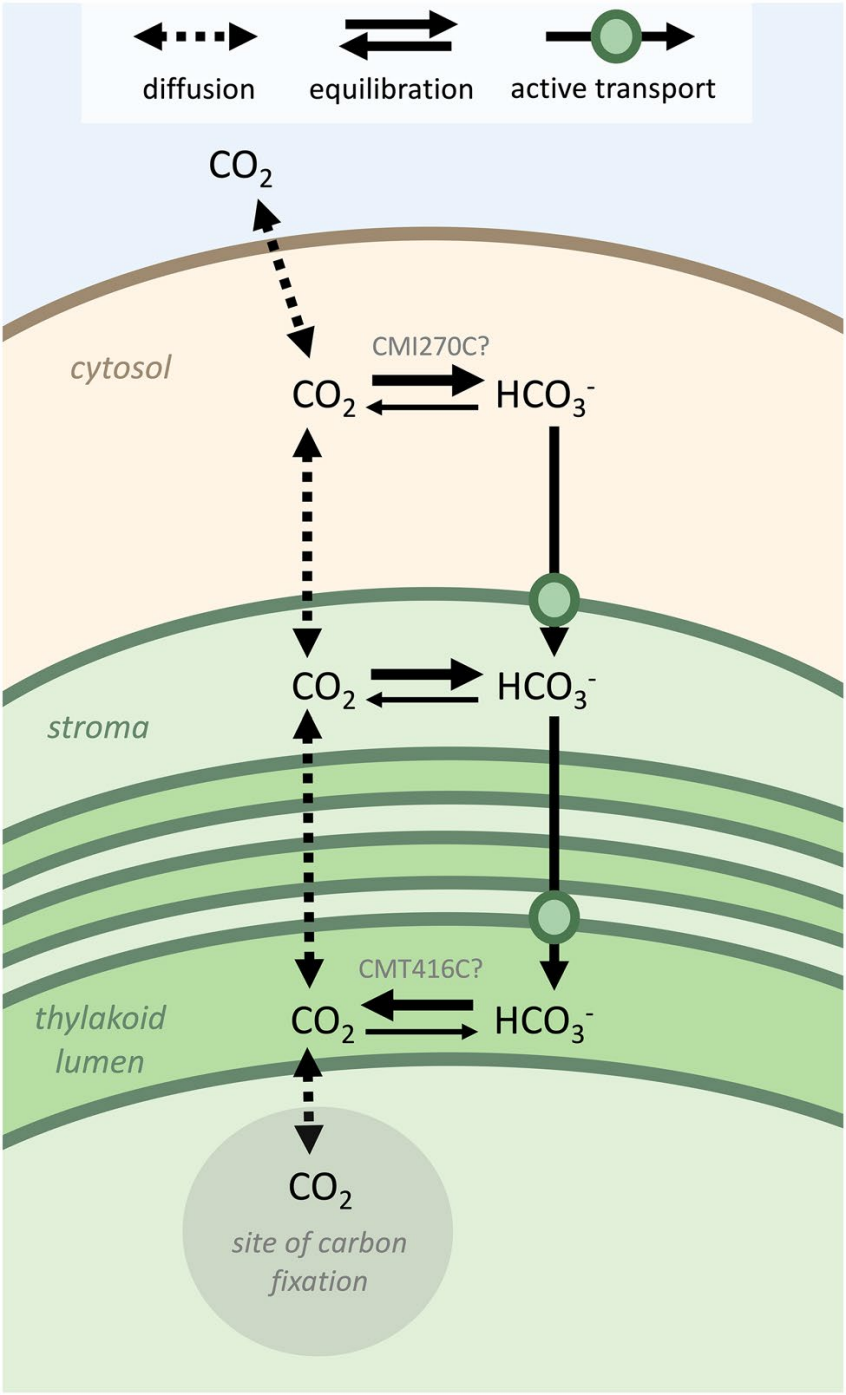
Hypothetical model of the CCM in *C. merolae*, indicating how Ci species may diffuse, interconvert, and be transported to concentrate carbon around the site of rubisco carboxylation (more details in text). In pairs of equilibration arrows, the thicker arrow points to the Ci species which is predicted to be more abundant in that compartment (see Fig. 4) (arrows are not to scale with relative abundances of Ci species). This image abbreviates the movement of Ci through the outer thylakoid rings, which we hypothesize may occur by active transport across thylakoids or by diffusion through discontinuities in the thylakoids. Carbonic acid may represent a minor membrane-permeating species alongside CO_2_

pH-gradient-based CCMs may be called “passive CCMs,” which refers to the primary mode of Ci entry into the cell, rather than a lack of protein or energy investment in the CCM. In fact, there are proteins which could facilitate the operation of a passive CCM and which have homologs in *C. merolae* (Table 3, Table S1). In our hypothetical model of *C. merolae*’s CCM (Fig. 5), active bicarbonate transporters at the chloroplast envelope and thylakoid membrane overcome the low membrane-permeability of bicarbonate (Mangan et al. 2016). There are several types of bicarbonate transporters which have homologs in *C. merolae* (Table 3), though this homology analysis is complicated by the fact that known bicarbonate transporters, like many CCM-involved genes, belong to widespread protein families whose members have diverse functions. In our analysis, it was common to obtain numerous hits for bicarbonate transporter queries, and reciprocal best hits analysis was subject to limitations of the NCBI database (e.g., inclusion of similar sequences from different experiments in combination with limited annotation of species and functions). In addition to bicarbonate transporters, a passive CCM would depend on maintenance of near-neutral pH in the cytosol, which in *C. merolae* requires a large investment in synthesizing and operating plasma membrane H^+^-ATPases. These proton pumps, which were found in one analysis to have the highest transcript abundance of any gene in *C. merolae*’s genome (Misumi et al. 2017), may consume up to 1 ATP per proton extruded into a low-pH extracellular environment (Zenvirth et al. 1985).

#### Carbonic anhydrases in the CCM

Carbonic anhydrase (CA) enzymes, which catalyze interconversion of Ci species, had homologs in *C. merolae*. CAs have diverse functions and not all CAs are involved in photosynthesis or a CCM, but these enzymes are part of the CCM in well-studied systems (Morita et al. 1998; Jensen et al. 2020). When a membrane-permeable CA inhibitor dissolved in DMSO was applied to *C. merolae* cells, cellular oxygen evolution did not decrease compared to DMSO alone (Moroney et al. 2004; Parys et al. 2021). However, the presence of CA homologs in *C. merolae*’s genome is consistent with the operation of a biophysical CCM. In particular, we identified two α-CA homologs which we hypothesize participate in the CCM (Table 2). These putative α-CAs are among the four *C. merolae* CA candidates which have homology to *Arabidopsis thaliana* CAs, and their localizations by fluorescence tagging and their transcriptional responses to CO_2_ availability have been previously discussed (Rademacher et al. 2017). The other two CA candidates previously discussed, CMM052C and CMD023C, (Rademacher et al. 2017) were identified by us as homologs to mitochondrial γ-CAs (Table S1), and since their localization predictions and annotations suggested mitochondrial functions, we did not include them in our model of the CCM.

CA localization and function invites further study in *C. merolae*, especially as the microalgal CCM is not as mechanistically well-understood as the cyanobacterial CCM, and molecular studies of microalgae other than *C. reinhardtii* are particularly sparse (Meyer and Griffiths 2013). It will be particularly useful to confirm where CAs function in the cell, as CCM function depends on proper CA localization. For example, the cyanobacterial CCM requires above-equilibrium bicarbonate concentrations in the cytosol and therefore depends on the absence of cytosolic CAs, while modeling of *C. reinhardtii*’s CCM suggests that CA distribution within an organelle would impact CCM function (Price and Badger 1989; Price et al. 2008; Fei et al. 2022). In *C. merolae*, a cytosolic CA, perhaps CMI270C (Rademacher et al. 2017), may be involved in cytosolic bicarbonate trapping. Another CA, perhaps CMT416C, may facilitate recapture of CO_2_ released during mitochondrial glycine decarboxylation of photorespiration. This role of CMT416C is supported by CMT416C’s predicted mitochondrial targeting sequence; by CMT416C’s fluorescence-tag localization between *C. merolae*’s mitochondrion and chloroplast; and by the existence of C_2_ photosynthesis, a plant carbon-concentrating mechanism which recaptures carbon from mitochondrial glycine decarboxylation (Sage et al. 2011; Rademacher et al. 2017). However, our model (Fig. 5) depicts an alternative function of CMT416C. CMT416C was predicted to have a chloroplast or thylakoid targeting sequence (Table 3) and thus we propose that CMT416C may be a thylakoid lumen enzyme with plastid import disrupted by fluorescence tagging. A thylakoid lumen CA, like CAH3 of *C. reinhardtii* (Moroney et al. 2011), may drive CO_2_ release around rubisco after Ci is pumped to above-equilibrium concentrations in the stroma. Although we used localization prediction tools trained on organisms evolutionarily distant from *C. merolae*, these tools’ plastidial localization predictions are often experimentally substantiated in *C. merolae*. For example, in *C. merolae*, plastidial localization predictions of acyl lipid metabolic enzymes from TargetP and PredAlgo are substantiated by fluorescence tagging in 72% (13/18) and 52% (14/27) of instances, respectively (Mori et al. 2016). TargetP plastidial localization predictions of *C. merolae*’s central carbohydrate metabolic enzymes are substantiated by fluorescence tagging in 89% (17/19) of instances (Moriyama et al. 2014). Tagging studies which engineer small additions onto carbonic anhydrases (i.e., epitope tag studies) or studies of functional complementation with fluorescence-tagged carbonic anhydrases would be useful to confirm these enzymes’ location of function.

#### Proposition of a biophysical CCM in *C. merolae*

We did not include C_4_ components in our CCM model, although the abundances of some C_4_ pathway components respond to carbon availability in *C. merolae* and *G. sulphuraria* (Rademacher et al. 2017; Curien et al. 2021). In *C. merolae*, transcripts of the C_4_ enzymes phosphoenolpyruvate carboxykinase (PEPCK), phosphoenolpyruvate carboxylase, and pyruvate phosphate dikinase increase in abundance following a shift from 5% CO_2_ to 0.04% CO_2_ conditions. Indeed, CCM components are often among those gene products upregulated by a shift to limiting CO_2_, and the evolution of C_4_ pathways may be relatively accessible and flexible, given the broad phylogenetic distribution of the necessary genes and the multiple evolutions of this pathway in plants (Hopkinson et al. 2016; Rademacher et al. 2017). However, the broad phylogenetic distribution of C_4_ genes also means that many organisms have C_4_ pathway components without a C_4_ CCM, and it is unclear whether single-cell C_4_ CCMs occur outside plants and macroalgae (von Caemmerer et al. 2014; Hopkinson et al. 2016; Jensen et al. 2020). *C. merolae*’s simple cellular structure may not permit an organizational structure analogous to the organelle partitioning that single-cell C_4_ CCMs use to fulfill the spatial regulation requirements of C_4_ photosynthesis (Edwards et al. 2004; Imoto and Yoshida 2017). Furthermore, a diffuse cytosolic localization of PEPCK in *C. merolae* (Moriyama et al. 2014) suggests that PEPCK decarboxylation is not directly involved in carbon concentration in this alga.

#### Chloroplast anatomy and the CCM

Unknown chloroplastic structural elements may also contribute to the CCM in *C. merolae*. Ultrastructural studies have not identified a recognizable pyrenoid in *C. merolae*, and this alga is described as lacking a pyrenoid (Broadwater and Scott 1994; Badger et al. 1998; Albertano et al. 2000; Misumi et al. 2005). Pyrenoids are membraneless organelles that support CCM function by aggregating rubisco into an environment conducive to efficient CO_2_ fixation, and the presence of a pyrenoid is highly correlated with the presence of a CCM in algae; very few algal species are known to maintain a CCM in the absence of a pyrenoid (Badger et al. 1998; Morita et al. 1998; Meyer and Griffiths 2013; Hennacy and Jonikas 2020; Barrett et al. 2021). We note that *C. merolae*’s lack of chloroplastic carbohydrate structures is not inconsistent with the presence of a pyrenoid. Like other organisms with red plastids (plastids which are in red algae or were acquired by endosymbiosis of red algae) *C. merolae* stores starch in the cytosol (Takusagawa et al. 2016; Toyoshima et al. 2016). Presumably as a result of this cytosolic starch storage, pyrenoids in red plastids apparently never have starch sheaths, except in cases where the pyrenoid is located in a chloroplast protrusion (Barrett et al. 2021). It is also not particularly informative that there was no identified *C. merolae* homolog to the *C. reinhardtii* rubisco linker that facilitates pyrenoid formation (Table S1). Proteins mediating the formation of membraneless organelles often have stretches of low-complexity sequences that may complicate homology analysis (Wunder et al. 2019; Barrett et al. 2021). *C. merolae* did have homologs to *C. reinhardtii* pyrenoid components (*C. reinhardtii* methyltransferases, a PSII subunit, a protein localized to mesh-like structures between pyrenoid starch plates, and enzymes of starch synthesis), though these components could have pyrenoid-unrelated functions in *C. merolae* (Table S1). Thus, localization of rubisco or carbon fixation will be necessary to resolve the local environment of *C. merolae*’s rubisco. Another chloroplast structure of interest is the thylakoids, as thylakoid membranes are candidates for diffusion barriers to CO_2_ (Barrett et al. 2021; Fei et al. 2022). *C. merolae*’s thylakoid membranes, which are arranged in concentric spheres and host arrays of very large light-harvesting complexes (Ichinose and Iwane 2017; Imoto and Yoshida 2017), may be particularly well-positioned to support a CCM.

### Gas-exchange results suggest the importance of further exploring photorespiration in CCM-containing organisms

In addition to an apparent lack of a pyrenoid*, C. merolae* has several other characteristics which may at first seem unusual for an organism with a CCM. Transitions to low-Ci environments are associated with increased cellular affinity for CO_2_ in some algae, presumably due to Ci-responsive expression of CCMs, but *C. merolae* cultures grown under 5% CO_2_ do not show a significantly lowered ${K}_{{m(CO}_{2})}$ of oxygen evolution when exposed to 0.04% CO_2_ conditions for 24 h (Spalding and Portis 1985; Badger et al. 1998; Giordano et al. 2005; Rademacher et al. 2016). However, *C. merolae* does have a transcriptional response to changes in CO_2_ availability, which includes shifts in the abundance of potentially CCM-involved transcripts (Rademacher et al. 2017). Even for the best-characterized algal CCM, that of the model alga *C. reinhardtii*, it is unclear how directly CCM physiology is tied to CO_2_ supply. CCM strength and molecular mechanisms in *C. reinhardtii* vary according to factors such as the intensity of CO_2_ limitation, the cellular division time in relation to the timing of environmental changes, and the presence of the photorespiratory byproduct and redox metabolite hydrogen peroxide (Vance and Spalding 2005; Mitchell et al. 2014; Wang and Spalding 2014; Neofotis et al. 2021). Furthermore*, C. reinhardtii*’s CCM is regulated by and requires light (Badger et al. 1980; Villarejo et al. 1996; Im and Grossman 2002), which suggests that induction of CCM physiology may be impacted by culture density and other conditions that are not directly related to CO_2_ availability. *C. merolae*, like the soil-dwelling alga *C. reinhardtii*, lives natively in a dynamic environment and may have a similarly versatile CCM.

Other arguments for the absence of the CCM in *C. merolae* focus on the apparent significance of photorespiration in this alga. However, the known photorespiratory characteristics of *C. merolae* are in fact compatible with a CCM. For example, one argument against a CCM in *C. merolae* is that its ${\Gamma }_{{CO}_{2}}$ is reduced to near-zero in 1.5% O_2_ conditions but is 60 ppm in 21% O_2_ conditions (Parys et al. 2021). One of the outcomes of all CCMs is indeed a minimized rubisco oxygenation reaction, and a ${\Gamma }_{{CO}_{2}}$ of 60 ppm at 21% O_2_ is indeed similar to the ~ 50 ppm reported for C_3_ plants at 20 °C (Tolbert et al. 1995; Giordano et al. 2005). However, *C. merolae*’s gas-phase ${\Gamma }_{{CO}_{2}}$ may not be directly comparable to the gas-phase ${\Gamma }_{{CO}_{2}}$ of plants characterized at moderate temperatures since this comparison does not account for the barriers to Ci acquisition and use in high-temperature aqueous environments. Our measurements and analysis indicated that *C. merolae*’s gas exchange physiology was quantitatively compatible with a CCM (Figs. 1, 2).

Another photorespiration-based argument against *C. merolae*’s CCM is that *C. merolae* knockouts of a photorespiratory glycolate oxidase have a high-Ci-requiring phenotype, which is attributed to high fluxes through the photorespiratory pathway. High photorespiratory fluxes in *C. merolae* would stand in contrast to the low photorespiratory fluxes traditionally associated with CCM-containing organisms (Rademacher et al. 2016). Additionally, glycolate oxidation by a glycolate oxidase, rather than by a glycolate dehydrogenase, is associated with an absent or inefficient CCM in cyanobacteria and some algae (Hagemann et al. 2016). However, the photorespiratory pathway is known to be essential in organism with CCMs, including C_4_ plants; CCM-containing algae; and cyanobacteria, which apparently always have CCMs (Raven et al. 2008; Moroney et al. 2013; Hagemann et al. 2016). Thus, necessity of the photorespiratory pathway cannot be diagnostic of an absent CCM. In fact, high glycolate oxidase activity is required for survival of the C_4_ plant maize in ambient air (Zelitch et al. 2008). *C. merolae*’s use of a photorespiratory glycolate oxidase could be explained by inefficiencies of the organism’s CCM (Table 2, Fig. 2), or by unique evolutionary factors influencing the development of *C. merolae*’s plant-type photorespiratory pathway. *C. merolae* has homologs to the nine enzymes of the *A. thaliana* photorespiratory pathway and to *A. thaliana* catalase, which detoxifies hydrogen peroxide produced by the photorespiratory pathway (Rademacher et al. 2016). Though *C. merolae* does not have close homologs to the plastidic dicarboxylate transporters which function in photorespiratory nitrogen recycling in plants, this may be explained by some flexibility in the localization of photorespiratory nitrogen metabolism across organisms, like the flexible localization observed for ammonium assimilation in seed plants (Barbier et al. 2005; Marino et al. 2022). Overall, rubisco oxygenation is present in all studied oxygenic photosynthetic organisms, and there are likely evolutionary barriers to eliminating this process (Moroney et al. 2013). Future studies may provide more clarity on the magnitude and role of photorespiratory processes in *C. merolae* and in other organisms which possess a CCM.

## Conclusion

We have described traits of *C. merolae* which are consistent with the operation of a CCM in this alga. Several aspects of this apparent CCM remain to be explored, including the CCM’s enzymatic and structural components and the role of photorespiration in this organism. Characterizing these features of *C. merolae* will further reveal how this extremophilic red alga survives in an environment which challenges photosynthesis.

## Methods

### Cell culture

A plate of *C. merolae* 10D cells was kindly provided by Dr. Peter Lammers (Arizona State University). Our cultures were started from liquid inocula at OD_750_ ~ 0.1 and were grown at 40 °C under 100 μmol m^−2^ s^−1^ continuous white light. Cells were grown as 50 mL cultures in 250 mL Erlenmeyer flasks, in media prepared according to a modified version of the Cyanidium Medium recipe from the Culture Collection of Algae at The University of Texas at Austin. This growth medium as prepared contained 3.78 mM (NH_4_)_2_SO_4_, 0.057 mM K_2_HPO_4_, 0.041 mM MgSO_4_ **·** 7H_2_0, 0.0015 mM CaCO_3_, and 1 mL solution per L media of Hutner’s Trace Elements. The medium was adjusted to pH 2.7 at room temperature by the addition of HCl. Cultures were aerated by shaking (50 rpm).

### Measurements of CO_2_ flux

Cells were harvested by spinning down ~ 5 mL culture samples (OD_750_ 1.2–1.7, 6–9 µg Chl *a *mL^−1^) at 300 × *g* for 10 min. Cells were then gently resuspended to 2 µg Chl *a* mL^−1^ (OD_750_ ~ 0.4, 15 mL samples) and loaded into the LI-6800 Aquatic Chamber (LI-COR Biosciences). The samples were resuspended in fresh growth medium prepared as described above, except for pH experiments, where samples were resuspended in growth medium with a pH of 2 at 40 °C, or in growth medium with a pH of 6 at 40 °C. To determine chlorophyll content for this resuspension, 1 mL cell samples were centrifuged (18,407 × *g*) for 5 min at room temperature. The cell pellet was then concentrated into 100 μL growth medium, and the concentrated cells were mixed by vortexing with 900 μL ice-cold methanol. After 30 min of dark incubation on ice, cell debris was pelleted out of the sample by centrifugation (18,407 × *g*) for 5 min at room temperature, and the absorbance of the resulting supernatant was analyzed on a Cary 60 UV–Vis spectrophotometer (Agilent Technologies). Like cyanobacteria, *C. merolae* does not have chlorophylls other than chlorophyll *a* (Cunningham et al. 2007). Thus, we used the spectrophotometric chlorophyll *a* concentration calculation equation published by Ritchie (2006) for cyanobacterial extracts in methanol, with a correction for extract turbidity at 720 nm.

The aquatic chamber temperature was maintained at 30 or 40 °C by use of a recirculating water bath and the instrument’s heat block temperature control function. Unless otherwise noted, the reference CO_2_ setpoint was 400 ppm, the light setpoint was 50% red 50% blue with 2000 μmol m^−2^ s^−1^ incident on the sample (~ 1100–1200 μmol m^−2^ s^−1^ absorbed by sample), and other environmental parameters were set as follows: flow of 400 μmol s^−1^, reference H_2_O control at least 20 mmol mol^−1^, fan speed of 14,000 rpm, subsample pump speed as set by 4.5 V direct current. The wait time between environmental condition changes and data logging was at least 480 s. Wait times were sufficient for fluxes to stabilize at least 1–2 min before logging, and exceeded the time needed for the media to adjust to changing CO_2_ concentrations (Fig. S3). The sample chamber was triple rinsed with deionized water between media or sample injections, and samples were examined under a light microscope after measurement to confirm the absence of contamination.

All parameters of interest were expressed in terms of headspace CO_2_ concentrations. Headspace CO_2_ concentrations were calculated using the concentration difference between the sample chamber CO_2_ concentration and headspace CO_2_ concentration ($\Delta {C}_{sub}$), as described in the manufacturer’s documentation: $\Delta {C}_{sub }= \frac{{\mu }_{i}}{{\mu }_{{i}_{sub}}}\Delta C$. $\Delta {C}_{sub}$ was calculated using the sample flow rate (${\mu }_{i}$), the subsample loop flow rate (${\mu }_{{i}_{sub}}$, measured as 233 μmol s^−1^), and the difference between the sample and reference chamber CO_2_ concentrations ($\Delta C$). To make the determination of headspace CO_2_ concentration using $\Delta {C}_{sub}$, we assumed that when reference chamber CO_2_ concentrations are higher than sample CO_2_ concentrations, sample chamber CO_2_ concentrations are higher than headspace CO_2_ concentrations. Negative headspace CO_2_ concentrations were replaced with zeroes before data analysis.

An averaging time of 19 s was used when logging data. Matching of the sample and reference analyzers was performed when the ΔCO_2_ < 10 ppm, if the reference chamber CO_2_ changed by > 100 ppm, or if the time between matches was > 30 min. In practice, this typically resulted in matching for each measured point.

During experiments examining the effect of oxygen concentration, a gas mixing system was used to introduce v/v mixes of 60% nitrogen / 40% oxygen, 79% nitrogen / 21% oxygen, or 98% nitrogen / 2% oxygen into the instrument at 1.5 standard liters per minute. The instrument’s CO_2_ injection system was then used to control CO_2_ abundance in the headspace. Samples were exposed to 21% oxygen conditions, then to 2% or 40% oxygen conditions, then again to 21% oxygen conditions to confirm that minimal shifting of photosynthetic fluxes had occurred during the experimental period.

Michaelis–Menten parameters (${K}_{{m(CO}_{2})}$, *A*_*max*_) were determined by using the R package “drc” (Version 3.0–1; Ritz et al. (2015)) to fit a two-parameter Michaelis–Menten equation to each replicate. CO_2_ compensation point was determined by fitting a linear trendline to CO_2_ response points ≤ 100 ppm CO_2_ in each replicate. Light respiration (*R*_*L*_) values were obtained by the Kok method, using the extrapolated intercept of a linear fit to points in each replicate with 10–30 μmol m^−2^ s^−1^ incident on the sample (~ 5.5–16.7 μmol m^−2^ s^−1^ absorbed by sample).

### Modeling gas-exchange parameters

Calculations were performed in R (R version 3.6.0). Parameter definitions, values, and sources not listed in the Methods are provided in Table 1.

CO_2_-O_2_ interactions in photosynthetic organisms and their impact on ${\Gamma }_{{CO}_{2}}$ are mechanistically well-understood, permitting the calculation of the CO_2_ compensation point ${\Gamma }_{{CO}_{2}}$ from rubisco kinetics and other information (von Caemmerer 2000):$\Gamma_{{CO_{2} }} = \frac{{\left( {0.5/S_{c/o} + \left( {K_{c} R_{L} } \right)/\left( {K_{o} V_{cmax} } \right)} \right)}}{{\left( {{\text{1 {-} }}R_{L} /V_{cmax} } \right)}}O + \frac{{K_{c} (R_{L} / V_{cmax} {) }}}{{{\text{(1 {-} }}R_{L} / V_{cmax} )}}$. Additionally, *K*_*c*_ values are of interest for comparison to the cellular${K}_{{m(CO}_{2})}$.

The kinetics of *C. merolae*’s rubisco are not known; recent collections of rubisco kinetics do not list kinetics for *C. merolae*’s rubisco (Young et al. 2016; Cummins et al. 2018; Flamholz et al. 2019), and to our knowledge these kinetics are not available elsewhere in the literature. Therefore, our calculation of ${\Gamma }_{{CO}_{2}}$ used all combinations of the reported rubisco kinetics of thermophilic Cyanidialean red algae closely related to *C. merolae* (see Miyagishima et al. (2017) for a rubisco-based phylogenetic tree of these organisms).

Reported rubisco kinetics were measured at 25 °C. However, we needed a quantitative framework to interpret gas-exchange parameters measured at 30 and 40 °C, as increasing temperatures challenge carboxylation by limiting rubisco’s CO_2_ affinity and specificity_*.*_ We therefore adjusted each kinetic parameter to *T* = 30 or 40 °C as in von Caemmerer (2000): $Parameter\left(T\right)=Parameter\left(25^\circ \mathrm{C}\right) {Q}_{10}^{\left[\frac{T-25}{10}\right]}$. To convert between the gas and liquid phase during temperature adjustments and elsewhere (calculating dissolved oxygen concentrations, converting physiological parameters from headspace concentrations to dissolved concentrations), we assumed an equilibrium defined by Henry’s law $C=HP$, where *C* is the concentration of a dissolved gas, *H* is the Henry’s law constant, and *P* is the gas partial pressure, which we calculated at an air pressure of 101,325 Pascal. Henry’s law constants vary with temperature, so the following equation was used to adjust the standard-temperature constants *H*_*298.15*_ to be appropriate for temperature *T* = 303.15 or 313.15 K according to $H\left(T\right)= {\text{H}}_{298.15}\mathrm{ exp}[\frac{-{\Delta }_{sol}H}{R}\left(\frac{1}{T}- \frac{1}{298.15}\right)]$. Henry’s law constants also vary according to the presence of other solutes in the solution, but this small effect is extremely difficult to estimate due to partially non-additive effects of solutes, and it is typically ignored by the rubisco community (Galmés et al. 2016). Some unit conversions required knowing the density of water at different temperatures, which we determined using a second-order polynomial fit to the water density values from Fierro (2007).

The rubisco *V*_*cmax*_ was estimated using the measured *A*_*max*_ of cell samples (Table 1; Fig. S1). *V*_*cmax*_ and *A*_*max*_ are comparable in this species: *C. merolae*’s rubisco *V*_*cmax*_ was measured in cell extracts (see below) as 27 pmol μg Chl *a*^−1^ s^−1^ at 25 °C, which is comparable to the cellular *A*_*max*_ of approximately 20 to 60 pmol μg Chl *a*^−1^ s^−1^ (in Fig. S1, 35 pmol μg Chl *a*^−1^ s^−1^ at 40 °C and 25 pmol μg Chl *a*^−1^ s^−1^ at 30 °C). *C. merolae*’s *A*_*max*_ and *V*_*cmax*_ are additionally comparable to *A*_*max*_ values of three green microalgae, which range from 0.90 to 1.8 pg C pg Chl *a*^*−*1^ h^−1^ (21 to 42 pmol μg Chl *a*^−1^ s^−1^) (Hupp et al. 2021).

The oxygen-response slope of the compensation point is also of interest as a physiological response influenced by the CCM, and can be extracted by fitting a linear trendline to compensation point predictions at various O_2_ concentrations. We additionally made comparisons to ${\Gamma }_{{CO}_{2}}$ data on the model alga *Chlamydomonas reinhardtii*, which operates a robust and well-characterized CCM (Mackinder 2017). Comparisons to *C. reinhardtii* were made by digitizing the compensation point data of Coleman and Colman (1980) with the WebPlotDigitizer application (v4.5, https://automeris.io/WebPlotDigitizer/). Following an ideal-gas-based unit conversion of the data on oxygen and temperature response of *C. reinhardtii*, we applied a linear regression to fit the data and used this regression to calculate the compensation point under conditions of interest. This digitizer application was also used to digitize some literature δ^13^C and *A*_*max*_ values.

### Dissolved Ci modeling

The R package “seacarb” (v2.1.12, Lavigne et al. (2019)) was used to calculate dissolved Ci concentrations under various environmental conditions.

### Rubisco activity assay

Dense cell cultures (OD_750_ ~ 2, ~ 50 mL) were concentrated (300 × *g*, 25 min) into 1 mL samples. These cell samples were washed (300 × *g*, 10 min) in growth media prepared at pH 7, and cell pellets were then placed at − 20 °C overnight. The pellets were then thawed in 1 mL extraction buffer (pH 8.1, 50 mM 3-[4-(2-Hydroxyethyl)piperazin-1-yl]propane-1-sulfonic acid (EPPS) buffer, 1% w/v polyvinyl polypyrrolidone, 1 mM ethylenediaminetetraacetic acid, 10 mM dithiothreitol, 0.1% Triton surfactant), then spun down (18,407 × *g*, 5 min) to remove cell debris and undissolved polyvinyl polypyrrolidone. To determine chlorophyll content for normalization of rubisco activity, 10 μL of extract was added to 990 μL methanol, and chlorophyll content was spectrophotometrically determined as described above.

To determine rubisco activity, the extract was tested by a spectrophotometric assay coupling reduced nicotinamide adenine dinucleotide (NADH) consumption to RuBP carboxylation, in a manner similar to the methods of Singh et al. (2022) and Kubien et al. (2010). The reaction was initiated by adding 10–80 μL extract and then 50 mM RuBP to a cuvette containing assay buffer (pH 8.0, 50 mM 2-[4-(2-Hydroxyethyl)piperazin-1-yl]ethane-1-sulfonic acid and sodium hydroxide (HEPES–NaOH) buffer, 20 mM magnesium chloride, 1 mM ethylenediaminetetraacetic acid, 1 mM adenosine triphosphate, 5 mM coupling enzyme cocktail, 20 mM sodium bicarbonate, 0.2 mM NADH). The coupling enzyme cocktail contained 20 units glyceraldehyde-3-phosphate dehydrogenase, 22.5 units 3-phosphoglyceric phosphokinase, 12.5 units creatine phosphokinase, 250 units carbonic anhydrase, and 56 units triose-phosphate isomerase. The rubisco carboxylation rate *V*_*cmax*_ was determined by monitoring the rate of RuBP-dependent NADH consumption at 340 nm and using NADH extinction coefficient 6.22 Abs_340_ mmol^−1^ cm^−1^ and carboxylation:NADH consumption stoichiometry of 1:4.

### Carbon isotope analysis

A gas analyzer (Aerodyne Research, Inc.) was used to determine δ^13^C of the ambient air with Tunable Infrared Laser Direct Absorption Spectroscopy. Raw gas analyzer output was corrected based on a calibration gas mixture of known isotopologues mixing ratio (Airgas, Inc.). Algae samples (OD_750_ 1.1, ~ 12 mL) were loaded into glass vials and freeze-dried in a FreeZone 12 lyophilizer (Labconco Corporation) at − 45 °C for approximately 27 h. The dried samples were ground into a fine powder and submitted for δ^13^C analysis to Lindsey Conaway and Erik Pollock (University of Arkansas Stable Isotope Laboratory). Biomass samples of approximately 0.3 mg were encapsulated in tin and analyzed on an EA-isolink elemental analyzer interfaced via ConFlo IV to a Delta V plus isotope ratio mass spectrometer (Thermo Electron Bermen). Raw measurements were normalized to international scale values using two reference materials: USGS41a (*n* = 3, standard δ^13^C = 36.55 ‰, measured δ^13^C = 36.55 ± 0.03 ‰ [mean ± S.D.]) and USGS8573 (*n* = 3, standard δ^13^C = − 26.39 ‰, measured δ^13^C = − 26.39 ± 0.09 ‰ [mean ± S.D.]).

### Identification of homologs to known CCM components

To identify *C. merolae* loci potentially involved in a carbon-concentrating mechanism, we used the BLASTX service of the *Cyanidioschyzon merolae* Genome Project (v3, http://czon.jp/blast/blast_cs.cgi). Queries were translated sequences of genes implicated in Ci accumulation by *C. reinhardtii,* bacteria, or diatoms (see Table S1 for a list of literature on these genes, which were sourced from Atkinson et al. (2015), Badger et al. (2002), Klanchui et al. (2017), Mackinder et al. (2017), Matsuda et al. (2017), Mukherjee et al. (2019), and Price et al. (2004)). BLASTP on the NCBI server (https://blast.ncbi.nlm.nih.gov/Blast.cgi) was used to determine whether apparent homologous proteins were reciprocal best hits. Subcellular localization of *C. merolae* loci was predicted with TargetP (Emanuelsson et al. 2000) and with PredAlgo when available (Tardif et al. 2012). Transcriptional data for *C. merolae* genes was sourced from Rademacher et al. (2017).

### Supplementary Information

Below is the link to the electronic supplementary material.

Supplementary file1 (PDF 1618 KB)

Supplementary file2 (XLSX 61 KB)

## Data Availability

The data and code have not been deposited in a public repository, but are available upon request through the corresponding author.
