# Correction to: The carbon-concentrating mechanism of the extremophilic red microalga *Cyanidioschyzon merolae*

**DOI:** 10.1007/s11120-023-01055-5

**Published:** 2023-10-18

**Authors:** Anne K. Steensma, Yair Shachar-Hill, Berkley J. Walker

**Affiliations:** 1 Department of Plant Biology, Michigan State University, East Lansing, MI USA; 2 Michigan State University - Department of Energy Plant Research Laboratory, Michigan State University, East Lansing, MI USA

**Correction to: Photosynthesis Research (2023) 156:247–264** 10.1007/s11120-023-01000-6

After publication, we discovered that an isotopically light CO_2_ source was leaking into our incubator during the time that cells were growing for δ^13^C analysis. As CCMs are associated with isotopically heavier δ^13^C signatures and supplementary CO_2_ may suppress CCMs or otherwise result in increased carbon isotope selectivity, we repeated the δ^13^C experiment with comparable methods and obtained a new *C. merolae* biomass δ^13^C value of − 19.75 ± 0.94‰ (mean ± 2 SEs) which is heavier than the previously reported value of − 23.03 ± 0.16‰. Our original conclusion of *C. merolae’s* biomass δ^13^C being consistent with a CCM is unaffected by this corrected result and even strengthened since the shift is to an isotopically heavier signature.

