# Supplementary material for: The carbon-concentrating mechanism of the extremophilic red microalga Cyanidioschyzon merolae
Source: Photosynth Res. 2023 Feb 13;156(2):247–64. doi: 10.1007/s11120-023-01000-6 (PMC10154280; doi:10.1007/s11120-023-01000-6)
Supplement: Supplementary file 1 — Supplementary file1 (PDF 1618 KB) [file 11120_2023_1000_MOESM1_ESM.pdf]

Supplementary Figures

Article Title

The Carbon-Concentrating Mechanism of the Extremophilic Red Microalga *Cyanidioschyzon merolae*

Author Information

Anne K Steensma[1,2]
Yair Shachar-Hill[1]
Berkley J Walker[1,2] (corresponding author, berkley@msu.edu)

[1] Michigan State University Department of Plant Biology, East Lansing, MI, USA
[2] Michigan State University-Department of Energy Plant Research Laboratory, East Lansing, MI, USA

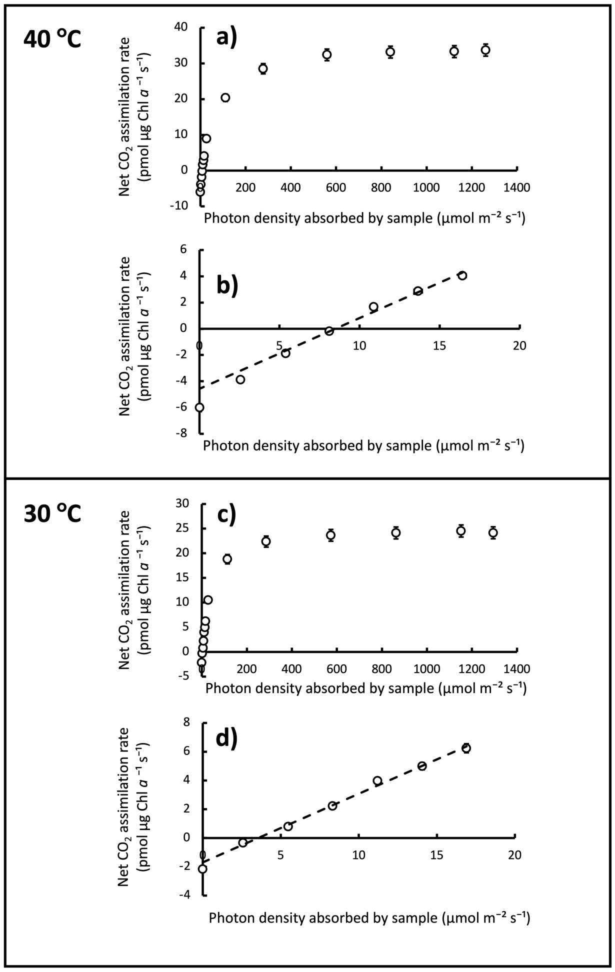

Fig. S1 Effect of absorbed light intensity on CO_2 assimilation of *C. merolae* at 40 °C **(a, b)** and 30 °C **(c, d)**. Panels **(a)** and **(c)** show the full light response curves, while panels **(b)** and **(d)** show the low-light region of the same data, with the linear trendline used to estimate R_L (dashed line). Points are means of n = 3, with error bars indicating ± 1 standard deviation of the x- and y-variables

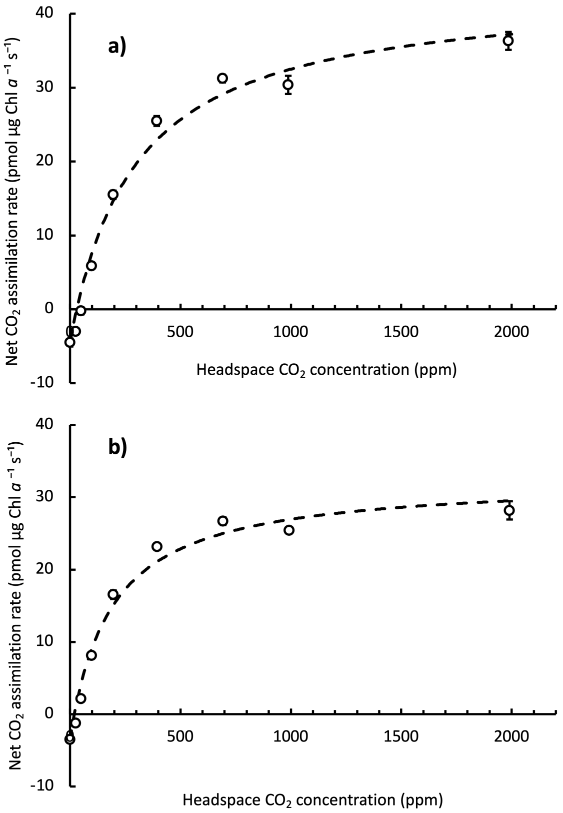

Fig. S2 Effect of CO_2 concentration on CO_2 assimilation of *C. merolae* at 40 °C **(a)** and 30 °C **(b)**, with curves defined by the average parameters from a Michaelis-Menten fit to each replicate (dashed lines) Points are means of n = 3, with error bars indicating ± 1 standard deviation of the x- and y-variables.

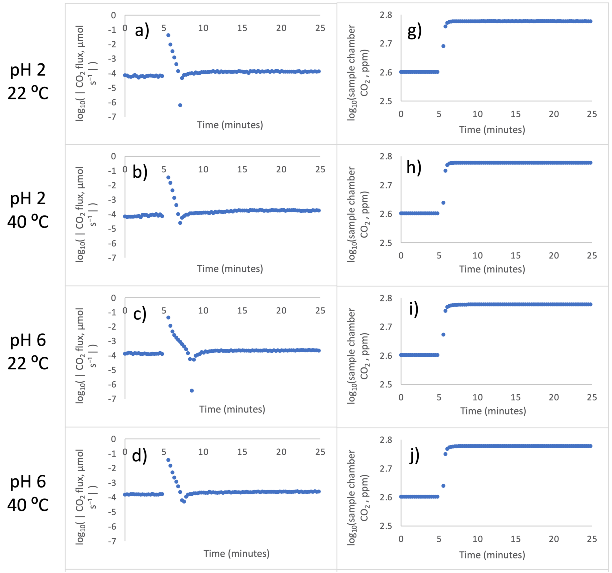

Fig. S3 Response of blank media to shifts in reference chamber CO_2 under varying pH and temperature conditions in the aquatic chamber. The response is presented in terms of CO_2 flux, with data converted to absolute values and $\log_{10}$-transformed before plotting **(a - f)**, or in terms of sample chamber CO_2 concentration, with data $\log_{10}$-transformed before plotting **(g - l)**
